# Time-Course of Redox Status, Redox-Related, and Mitochondrial-Dynamics-Related Gene Expression after an Acute Bout of Different Physical Exercise Protocols

**DOI:** 10.3390/life12122113

**Published:** 2022-12-15

**Authors:** Ramon Alves Pires, Thiago Macedo Lopes Correia, Amanda Alves Almeida, Raildo da Silva Coqueiro, Marco Machado, Mauro Fernandes Teles, Álbert Souza Peixoto, Raphael Ferreira Queiroz, Rafael Pereira

**Affiliations:** 1 Integrative Physiology Research Center, Department of Biological Sciences, Universidade Estadual do Sudoeste da Bahia (UESB), Jequie 45210-506, Brazil; 2 Multicentric Postgraduate Program in Biochemistry and Molecular (Brazilian Society for Biochemistry and Molecular Biology), Universidade Estadual do Sudoeste da Bahia (UESB), Vitoria da Conquista, Jequie 45210-506, Brazil; 3 Multicentric Postgraduate Program in Physiological Sciences (Brazilian Society of Physiology), Universidade Federal da Bahia (UFBA), Vitoria da Conquista, Jequie 45210-506, Brazil; 4 Fundação Universitária de Itaperuna (FUNITA), Itaperuna 28300-000, Brazil; 5 Laboratory of Physiology and Biokinetic, Faculty of Biological Sciences and Health, Universidade Iguaçu Campus V, Itaperuna 28300-000, Brazil; 6 Instituto de Ciências Biomédicas (ICB), Universidade de São Paulo (USP), São Paulo 05508-000, Brazil; 7 Postgraduate Program in Biosciences, Universidade Federal da Bahia, Campus Anísio Teixeira, Vitória da Conquista 40110-100, Brazil

**Keywords:** exercise, mitochondrial biogenesis, mitochondrial fusion, mitochondrial fission, mitophagy, oxidative stress

## Abstract

We investigated the magnitude of exercise-induced changes in muscular bioenergetics, redox balance, mitochondrial function, and gene expression within 24 h after the exercise bouts performed with different intensities, durations, and execution modes (continuous or with intervals). Sixty-five male Swiss mice were divided into four groups: one control (n = 5) and three experimental groups (20 animals/group), submitted to a forced swimming bout with an additional load (% of animal weight): low-intensity continuous (LIC), high-intensity continuous (HIC), and high-intensity interval (HII). Five animals from each group were euthanized at 0 h, 6 h, 12 h, and 24 h postexercise. Gastrocnemius muscle was removed to analyze the expression of genes involved in mitochondrial biogenesis (*Ppargc1a*), fusion (*Mfn2*), fission (*Dnm1L*), and mitophagy (*Park2*), as well as inflammation (*Nos2*) and antioxidant defense (*Nfe2l2*, *GPx1*). Lipid peroxidation (TBARS), total peroxidase, glutathione peroxidase (GPx), and citrate synthase (CS) activity were also measured. Lactacidemia was measured from a blood sample obtained immediately postexercise. Lactacidemia was higher the higher the exercise intensity (LIC < HIC < HII), while the inverse was observed for TBARS levels. The CS activity was higher in the HII group than the other groups. The antioxidant activity was higher 24 h postexercise in all groups compared to the control and greater in the HII group than the LIC and HIC groups. The gene expression profile exhibited a particular profile for each exercise protocol, but with some similarities between the LIC and HII groups. Taken together, these results suggest that the intervals applied to high-intensity exercise seem to minimize the signs of oxidative damage and drive the mitochondrial dynamics to maintain the mitochondrial network, similar to low-intensity continuous exercise.

## 1. Introduction

Antioxidant defense and mitochondrial dynamic improvements are two of the main benefits of physical training [1]. Indeed, the increase of metabolic demands observed during and immediately after a physical exercise bout induces changes in the molecular machinery of the muscle cells, which include changes in the gene expression patterns, leading to the up- or downregulation of different target genes in the first hours after the exercise bout [1,2].

These acute exercise-induced molecular pattern changes are determinant of chronic adaptations, since it is the basis of protein synthesis, as molecular motors (e.g., myosin II), antioxidant enzymes, and regulatory proteins of mitochondrial dynamics (e.g., PGC-1α, mitofusins 1 and 2, dynamin-related protein 1, Parkin, and others) [2,3,4]. On the other hand, the molecular pattern depends on the kind and magnitude of stimulus generated by an exercise bout [2,5].

Exercise sessions characterized by low intensity and long duration are recognized as an adequate design to induce better metabolic adaptations [1]. However, studies investigating the effect of high-intensity interval training (HIIT) have consistently reported great metabolic adaptations, many of them at a similar magnitude to low-intensity and long-duration training [2], despite the different stimuli.

In fact, at a proper mode, intensity, and duration, exercise bouts induce metabolites (e.g., lactate) and reactive-oxygen-species (ROS) generation within the muscle cells, which plays an essential role in regulating a wide range of biological functions that directly or indirectly affect these adaptations [4,6]. A recent debate has arisen regarding concern about the deleterious effects of high-intensity exercises, especially in the field of mitochondrial structure and function, owing to the generation of a harmful redox imbalance [2,3,7,8].

Despite the increasing debate and recent experimental data, there is no previous study comparing more than two experimental exercise protocols simultaneously. Previous studies are focused on comparing exercise protocols with different intensities (e.g., high-intensity and low/moderate-intensity exercise protocols) or with different execution designs (e.g., continuous and interval exercise protocols) and durations (e.g., short, and long duration). Thus, the present study aimed to investigate the redox status, redox-related, and mitochondrial-dynamics-related gene expression in the first 24 h after an acute bout of low-intensity-continuous-, high-intensity-continuous-, and high-intensity-intermittent-exercise protocols.

## 2. Methods

### 2.1. Animals

Male Swiss mice (n = 65), 12 weeks old, were purchased from Anilab (Paulínia, SP, Brazil). Animals were housed in polycarbonate cages and maintained under a 12 h light/dark cycle at a standard room temperature (23 ± 3 °C) and ad libitum access to water and commercial standard chow were provided.

All experimental procedures were conducted following the National Institutes of Health (NIH) Guide for the Care and Use of Laboratory Animals and approved by the Animal Experimentation Committee of the Universidade Estadual do Sudoeste da Bahia (protocol n° 175/2018).

### 2.2. Exercise Protocol

**Figure 1:**
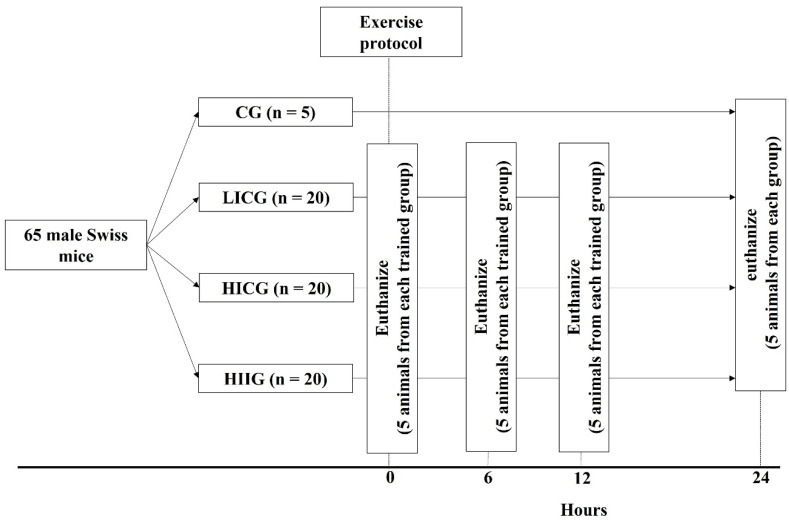
Experimental design.

Initially, the animals were exposed to 10 min of familiarization with the aquatic environment for 5 consecutive days. The animals were kept in the glass aquarium with the water at the level of their paws. Two days after the familiarization period, the animals were randomly divided into four groups: (1) the control group (CG; n = 5); (2) the low-intensity-continuous-exercise group (LIC; n = 20), which was submitted to a forced swim for 30 min with an additional load, corresponding to 2.5% of each animal weight; (3) the high-intensity-continuous-exercise group (HIC; n = 20), which was submitted to a forced swim for 15 min with an additional load, corresponding to 5% of each animal weight; (4) the high-intensity-interval-exercise group (HII; n = 20), which was submitted to a forced swim for 7 min (14 cycles of 20 s swimming/10 s resting) with an additional load, corresponding to 10% of each animal weight (see Figure 1). A bag with small lead spheres was attached to the tail base of each animal. The weight of each animal was measured using a digital scale (VL-3200H, Shimadzu, Lenexa, KS, USA) and the workload was adjusted for each animal to achieve the proposed load. The used exercise protocols allowed a similar session volume between the LIC and HIC groups (1800 s × 2.5% of animals weight = 4500; 900 s × 5% of animals weight = 4500), but a 38% lower session volume for the HII group (280 s × 10% of animals weight = 2800).

Mice from exercised groups (i.e., LIC, HIC, and HII) were placed in a swimming apparatus described previously [9,10] with adaptations, consisting of a glass aquarium (35 cm high, 35 cm wide, and 50 cm long) divided into 2 lanes (15 × 15 cm per lane and a depth of 35 cm) to allow individual training. The water temperature was kept constant (32–34 °C) during all experimental procedures. Animals from the HII group were taken from the water with a steel skimmer, maintained resting for 10 s over the steel skimmer, and then put back in the water.

Immediately after the exercise protocol, 5 animals from each exercised group were anesthetized with intraperitoneal administration of xylazine (16 mg/kg) and ketamine (50 mg/kg), and then euthanized by decapitation. Trunk blood was collected in 1.5 mL dry microtubes and centrifuged (14,000 rpm at 4 °C for 15 min) for serum separation and was stored in a −70 °C freezer until the serum lactate measurement. Gastrocnemius muscles of both hind limbs were dissected, weighed, identified, frozen in liquid nitrogen, and stored in a −70 °C freezer for biochemical and molecular biology assays. Five animals from each exercised group were euthanized, following the same described procedures, at 6, 12, and 24 h after the exercise protocol, while the 5 animals from CG were euthanized only at 24 h after the exercise protocol.

Serum lactate was measured by the automated assay method using a monoreagent colorimetric enzymatic kit (lactate oxidase 400 U/L and peroxidase (Horseradish) 2400 U/L) (ABBOTT, Decatur, TX, USA). This parameter was measured only in the blood samples obtained immediately after the exercise protocol, since the serum lactate trend is to return to basal level some minutes after any exercise. The serum lactate was used as an estimative of the exercise intensity to compare the proposed exercise protocols.

### 2.3. Determination of Total Peroxidase and Glutathione Peroxidase Activities in Muscle Samples

For biochemical assays, muscle tissue (100 mg/mL) was homogenized in RIPA buffer (Sigma-Aldrich, Saint Louis, MO, USA) and centrifuged at 1600× *g* for 10 min at 4 °C (Z 36 HK, Hermle-Labortechnik, Wehingen, Germany). Supernate was collected for determining the thiobarbituric-acid-reactive substances (TBARS), total peroxidase, and glutathione peroxidase activities.

TBARS levels were calculated based on the standard curve of malondialdehyde (MDA) (Cayman Chemical, Ann Arbor, MI, USA) (0 to 50 µM) and followed the protocol described previously [11]. TBARS levels (μM) in muscle tissue were normalized by protein concentration (mg/mL) and expressed as μM/mg protein.

Total peroxidase activity was measured by the disappearance of hydrogen peroxide (H_2_O_2_) at 240 nm [12], whilst the total glutathione peroxidase (GPx) activity was determined according to previously standardized methods [13]. Total peroxidase and GPx activities were normalized by protein concentration (mg/mL) and expressed in mU/mg protein.

Total protein concentration of the muscle homogenates was determined by Bradford colorimetric assay at 595 nm (Sigma-Aldrich, Saint Louis, MO, USA) employing serum bovine albumin (Sigma-Aldrich, Saint Louis, MO, USA) (0 to 1.4 mg/mL) as standard [14].

### 2.4. Determination of Citrate Synthase (CS) Activity

Citrate synthase activity was measured using a microplate spectrophotometer. Muscle tissue was homogenized as described for total peroxidase and GPx assays and the citrate synthase activity was measured as described previously [15].

### 2.5. Quantitation of mRNA by RT-qPCR

Total RNA was isolated from gastrocnemius muscles using the TRIzolTM reagent (Invitrogen, MA, USA) and the TissueRuptor system (Qiagen, Germantown, MD, USA) was used for the muscle tissue homogenization. Complementary cDNA was synthesized from 2 µg of total RNA using a High-Capacity RNA-to-cDNA^TM^ reverse transcription kit (Applied Biosystem, Bedford, MA, USA).

For quantitative real-time polymerase chain reaction (qPCR), the TaqMan^®^ Fast Advanced Master Mix System (Applied Biosystem, Bedford, MA, USA) was used to examine the messenger RNA (mRNA) expression levels of seven target genes involved in mitochondrial dynamics: *Ppargc1α*, *Mfn2*, *Dnm1L*, *Park2 Nfe2l2, Gpx1,* and *Nos2*, encoding the proteins: peroxisome-proliferator-activated receptor gamma coactivator 1-alpha (PGC-1α), mitofusin-2 (MFN2), dynamin-related protein 1 (Drp1) and Parkin, nuclear factor erythroid 2-related factor (Nrf2), glutathione peroxidase 1 (GPX1), and inducible nitric oxide synthase (NOS2), respectively (see Table 1). All procedures were performed according to the manufacturer’s instructions, and thermocycling was performed using the StepOne Plus thermal cycler (Applied Biosystem, Bedford, MA, USA). Gene encoding the constitutive protein GAPDH (glyceraldehyde-3-phosphate dehydrogenase) (see Table 1) was amplified as a housekeeping gene. Gene expression results were presented as a relative expression (fold change) and calculated using the comparative method (2^−ΔΔCt^), as proposed previously [16].

### 2.6. Statistical Analysis

All variables of interest in this study were submitted to the Shapiro–Wilk normality test. The lactate data were log-transformed to normalize its distribution. For statistical analysis purposes, comparisons were made with time intervals after exercise (0 h (immediately after), 6 h, 12 h, and 24 h) within each group and between the 3 groups (LIC vs HIC vs HII) at each time interval. Comparisons between groups were performed using one-way ANOVA, followed by Tukey’s post hoc test, adopting a significance level of *p* ≤ 0.05 for all tests. Data were presented as mean ± standard error (SE) and all statistical procedures were performed using the GraphPad Prism 7.0 software (La Jolla, CA, USA).

## 3. Results

### 3.1. Serum Lactate, TBARS, and Enzymatic Activity (Total Perosidase, GPx, and CS)

**Figure 2:**
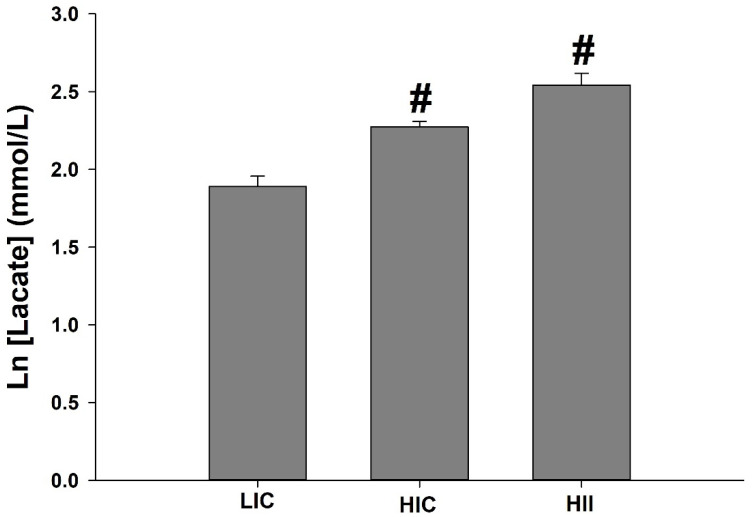
Mean ± SE of serum lactate immediately (0 h) after the low-intensity-continuous (LIC), high-intensity-continuous (HIC), and high-intensity-interval (HII) exercise bouts. One-way ANOVA indicated between-group differences. (#) Significantly different from other groups (LIC < HIC < HII); (*p* < 0.05).

The blood lactate was significantly higher in the HII group when compared to the other groups, while the HIC group also presented significantly higher values than the LIC group (Figure 2).

**Figure 3:**
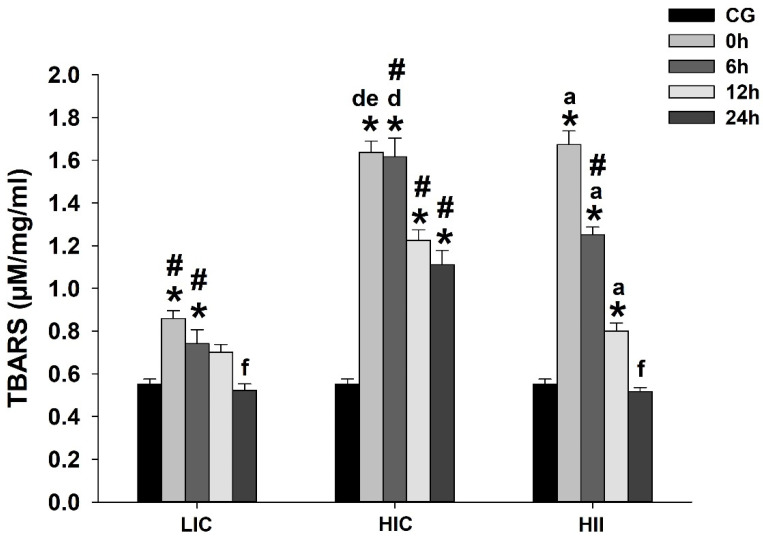
Mean ± SE of thiobarbituric-acid-reactive substances (TBARS) levels in gastrocnemius muscle from mice exercised with low-intensity-continuous (LIC), high-intensity-continuous (HIC), and high-intensity-interval (HII) exercise obtained at 0 h (i.e., immediately), 6 h, 12 h, and 24 h postexercise. TBARS levels from the control group (CG) were obtained from nonexercised mice. One-way ANOVA indicated between-group differences. (*) Significantly different from CG; (a) Significantly different from other times in the same group; (d) Significantly different from 24 h in the same group; (e) Significantly different from 12 h in the same group; (f) Significantly different from 0 h, 6 h, and 12 h in the same group; (#) Significantly different from the other groups at the same time; (*p* < 0.05).

The TBARS levels from the LIC group were significantly higher than the CG immediately (i.e., 0 h) and 6 h postexercise (*p* < 0.05), declining progressively with time, with significantly lower levels at 24 h postexercise (*p* < 0.05) when compared to all other measures from the same group. The HIC group exhibited significantly higher TBARS levels at 0 h and 6 h (*p* < 0.05) when compared to the CG and LIC group. These higher levels at 0 h were sustained at 6 h, with a significant difference from the LIC and HII groups (*p* < 0.05). At 12 h to 24 h postexercise, the TBARS levels from the HIC group declined significantly when compared to 0 h and 6 h from the same group (*p* < 0.05), but still significantly higher than all other groups at the same times (i.e., 12 and 24 h) (*p* < 0.05). As with the HIC group, the HII group exhibited higher TBARS levels immediately after the exercise bout, with a significant difference from the CG and LIC group (*p* < 0.05), but it declined progressively from 6 h to 24 h after the exercise bout. In the HII group, the measure at 6 h remained significantly higher than the CG and LIC group (*p* < 0.05), and the measure at 12 h remained significantly higher only in the CG (*p* < 0.05). At 24 h, for the HII group, the TBARS levels declined to levels observed in the CG and LIC group, with significantly lower levels than all other measures from the same group (*p* < 0.05). Figure 3 presents the TBARS levels from each group at each measure over time.

**Figure 4:**
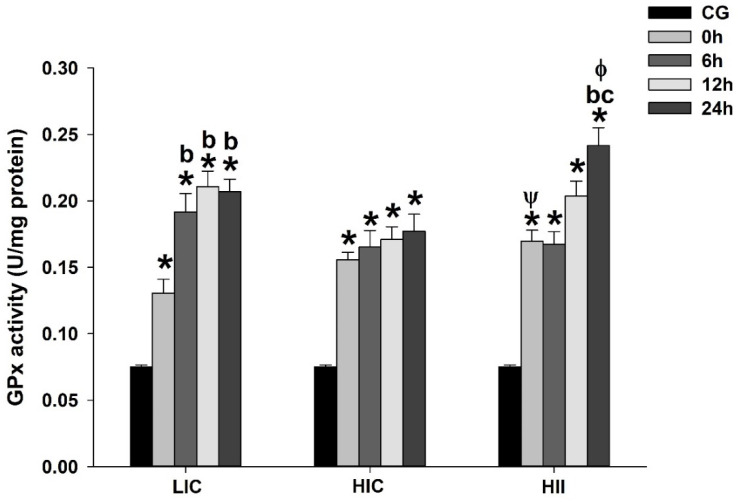
Mean ± SE of total GPx activity in gastrocnemius muscle from mice exercised with low-intensity-continuous (LIC), high-intensity-continuous (HIC), and high-intensity-interval (HII) exercise obtained at 0 h (i.e., immediately), 6 h, 12 h, and 24 h postexercise. GPx activity from the control group (CG) was obtained from nonexercised mice. One-way ANOVA indicated between-group differences. (*) Significantly different from CG; (b) Significantly different from 0 h in the same group; (c) Significantly different from 6 h within the same group; (Ψ) Significantly different from LIC at the same time; (Φ) Significantly different from HIC at the same time; (*p* < 0.05).

Compared to the CG, the total GPx activity was significantly higher up to 24 h after the exercise bout in all exercised groups (*p* < 0.05). All exercised groups exhibited a progressive increase of the total GPx activity over time (i.e., 0 h to 24 h) after the exercise bout, but the HII group exhibited significantly higher total GPx activity when compared to the LIC group at 0 h and the HIC group at 24 h after the exercise bout. The HIC group was the unique exercised group without a significant difference in within-group comparisons. However, the LIC group exhibited significantly higher total GPx activity from 6 h to 24 h when compared to 0 h from the same group, while the HII group exhibited significantly higher total GPx activity at 24 h postexercise when compared to 0 h and 6 h from the same group. Figure 4 presents the total GPx activity from each group at each measure over time.

**Figure 5:**
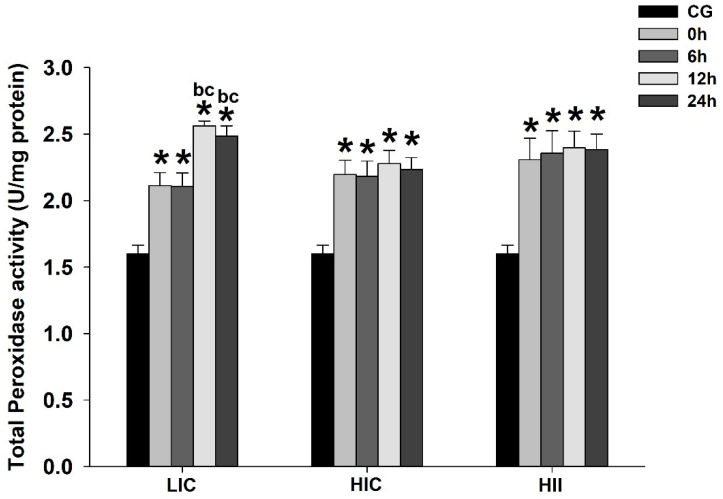
Mean ± SE of total peroxidase activity in gastrocnemius muscle from mice exercised with low-intensity-continuous (LIC), high-intensity-continuous (HIC), and high-intensity-interval (HII) exercise obtained at 0 h (i.e., immediately), 6 h, 12 h, and 24 h postexercise. Total peroxidase activity from the control group (CG) was obtained from nonexercised mice. One-way ANOVA indicated between-group differences. (*) Significantly different from CG; (b) Significantly different from 0 h in the same group; (c) Significantly different from 6 h within the same group (*p* < 0.05).

Compared to the CG, the total peroxidase activity was significantly higher in all exercised groups at all measures after exercise in all studied groups. Significant differences were not observed between the exercised groups. Only the LIC group exhibited a significant difference in the within-group comparisons, with a significantly higher total peroxidase activity observed at 12 h and 24 h postexercise when compared to measurements at 0 h and 6 h (*p* < 0.05) (Figure 5).

**Figure 6:**
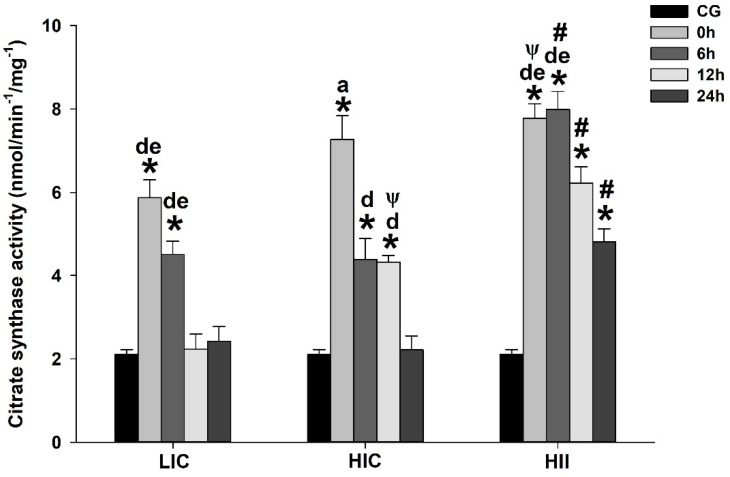
Mean ± SE of citrate synthase activity (CS) in gastrocnemius muscle from mice exercised with low-intensity-continuous (LIC), high-intensity-continuous (HIC), and high-intensity-interval (HII) exercise obtained at 0 h (i.e., immediately), 6 h, 12 h, and 24 h postexercise. CS activity from the control group (CG) was obtained from nonexercised mice. One-way ANOVA indicated between-group differences. (*) Significantly different from CG; (a) Significantly different from other moments in the same group; (d) Significantly different from 24 h in the same group; (e) Significantly different from 12 h in the same group; (Ψ) Significantly different from LIC at the same time; (#) Significantly different from the other groups at the same time; (*p* < 0.05).

All groups exhibited a peak of CS activity immediately after the exercise bout, declining along the measures obtained up to 24 h postexercise. Compared to the CG, the CS activity was significantly higher in all exercised groups at 0 h and 6 h, while in the HIC and HII groups, it was also significantly higher at 12 h, remaining higher until 24 h after the exercise bout only in the HII group (*p* < 0.05). The comparisons among the exercised groups indicated higher CS activity in the HII group than in the LIC group immediately postexercise bout (*p* < 0.05). Additionally, from 6 h to 24 h after the exercise bout, animals submitted to HII exercise exhibited higher CS activity than all other exercised groups (*p* < 0.05), while the animals submitted to HIC exercise exhibited higher CS activity than for LIC exercise at 12 h after the exercise bout (*p* < 0.05) (Figure 6).

Within-group comparisons indicated higher CS activity in the LIC group at 0 h and 6 h postexercise when compared to measures obtained at 12 h and 24 h (*p* < 0.05). The CS activity immediately after the exercise bout was higher than all other measures from the HIC group, while the measures at 6 and 12 h were significantly higher than 24 h postexercise (*p* < 0.05). The HII group exhibited higher CS activity at 0 h and 6 h postexercise when compared to the measures obtained at 12 h and 24 h (*p* < 0.05) (Figure 6).

### 3.2. Mitochondrial Dynamics-Related Gene Expression in Muscle Tissue Samples

**Figure 7:**
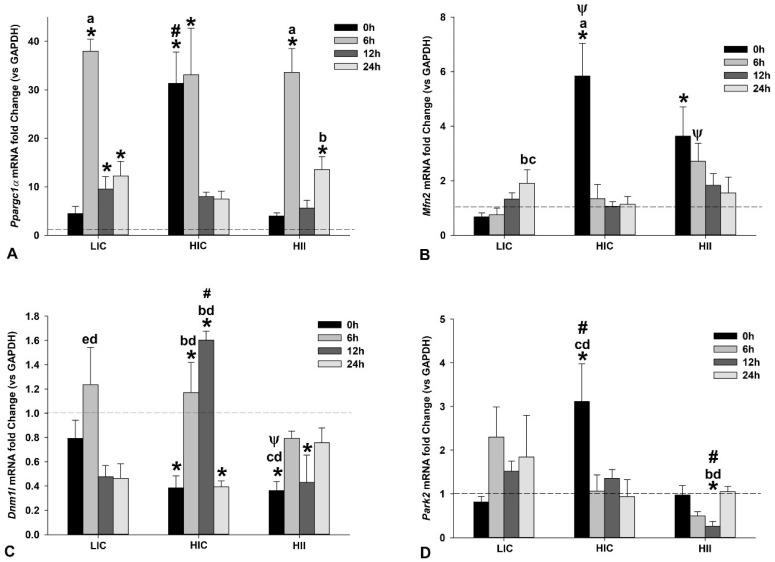
Presentation of target genes involved in mitochondrial dynamics. Mean ± SE of gene expression of *Ppargc1α* (**A**), *Mfn2* (**B**), *Dnm1l* (**C**), and *Park2* (**D**) in gastrocnemius muscle from mice exercised with low-intensity-continuous (LIC), high-intensity-continuous (HIC), and high-intensity-interval (HII) exercise obtained at 0 h (i.e., immediately), 6 h, 12 h, and 24 h postexercise. Gene expression from the control group (CG) was obtained from nonexercised mice and is represented by the horizontal dashed line. One-way ANOVA indicated within-group and between-group differences. (*) Significantly different from CG; (a) Significantly different from other moments in the same group; (b) Significantly different from 0 h in the same group; (c) Significantly different from 6 h within the same group; (d) Significantly different from 24 h in the same group; (e) Significantly different from 12 h in the same group; (Ψ) Significantly different from LIC at the same time; (#) Significantly different from the other groups at the same time; (*p* < 0.05).

*Ppargc1α* gene expression in the LIC group was significantly higher at 6, 12, and 24 h after the exercise bout when compared to the CG. The peak of gene expression in the LIC group was observed 6 h after the exercise bout, which was significantly higher than all other measures from the same group (*p* < 0.05). Differently, in the HIC group, the peak of gene expression was observed immediately and sustained at 6 h after the exercise bout, being significantly higher than the CG and the measures obtained at 12 h and 24 h from the same group. The *Ppargc1α* gene expression in the HIC group obtained immediately after the exercise bout was significantly higher than in other groups at the same time (i.e., 0 h). The Ppargc1α gene expression in the HII group exhibited a behavior close to the LIC group, with a peak at 6 h after the exercise bout, which was significantly higher than the CG and all other measures from the same group. At 24 h after the exercise bout, the gene expression was also higher than the CG and the measure obtained immediately after the exercise bout from the same group. Figure 7A presents the results from the Ppargc1α gene expression.

*Mfn2* gene expression in the LIC group increased along the studied time and achieved significantly higher levels at 24 h when compared to 0 h and 6 h after the exercise bout from the same group. Differently, both high-intensity groups (i.e., HIC and HII) exhibited a peak of *Mfn2* gene expression immediately (i.e., 0 h) after the exercise bout, declining along the studied time. In both high-intensity groups, the *Mfn2* gene expression at 0 h was significantly higher than the CG, while in the HIC group it was also higher than all other measures from the same group as well as for the LIC group at the same time (i.e., 0 h). On the other hand, *Mfn2* gene expression was significantly higher in the HII group at 6 h after the exercise bout than for the LIC group at the same time. Figure 7B presents the results from the *Mfn2* gene expression.

Despite a trend to repression, *Dnm1l* gene expression in the LIC group was not statistically different from the CG, but a peak of gene expression was observed at 6 h after the exercise bout, which was significantly different from 12 and 24 h from the same group. In the HIC group, the *Dnm1l* gene expression exhibited two distinct behaviors: a trend to repression at 0 h and 24 h postexercise, with significantly lower gene expression than the CG, and increased gene expression at 6 h and 12 h, which were statistically significantly higher than the CG, as well as the measures at 0 h and 24 h from the same group. The HII group was the unique group, with a clear trend to the repression of the *Dnm1l* gene expression, which was significantly lower than the CG immediately and 12 h postexercise. Additionally, the *Dnm1l* gene expression was significantly lower in the HII group at 0 h than for the LIC group at the same time, and at 6 h and 24 h from the same group. Figure 7C presents the results from the *Dnm1l* gene expression.

*Park2* gene expression exhibited a divergent trend among the studied groups. In the LIC group, the *Park2* gene expression exhibited a trend to increase from 6 h to 24 h after the exercise bout but without a significant difference to the CG (*p* > 0.05). The *Park2* gene expression immediately postexercise was significantly higher in the HIC group than the CG, all other groups at the same time, and 6 h and 24 h from the same group. The HII group was the unique group, with a clear trend to the repression of the *Park2* gene expression after the exercise bout, achieving a significantly lower level at 12 h postexercise when compared to the CG and all other groups at the same time. Additionally, it was significantly lower at 12 h than 0 h and 24 h from the same group. Figure 7D presents the results from the *Park2* gene expression.

### 3.3. Redox-Related Gene Expression in Muscle Tissue Samples

**Figure 8:**
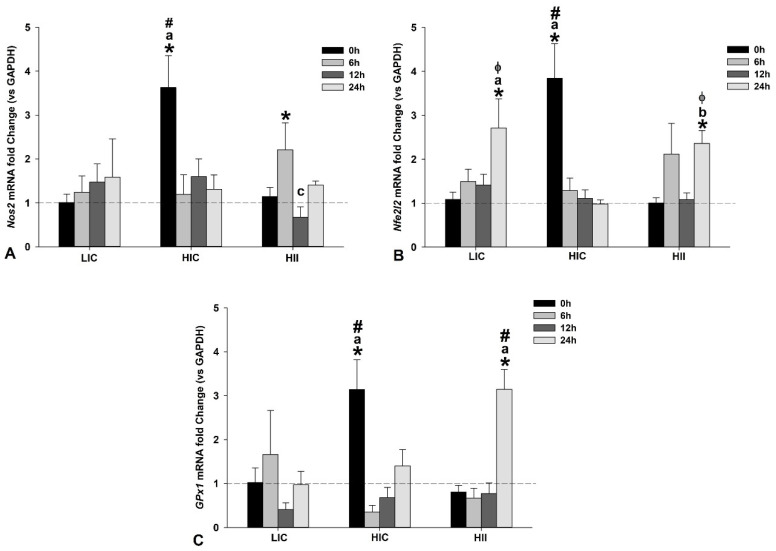
Presentation of target genes involved in redox balance. Mean ± SE of gene expression of *Nos2* (**A**), *Nfe2l2* (**B**), and *GPx1* (**C**) in gastrocnemius muscle from mice exercised with low-intensity-continuous (LIC), high-intensity-continuous (HIC), and high-intensity-interval (HII) exercise obtained at 0 h (i.e., immediately), 6 h, 12 h, and 24 h postexercise. Gene expression from the control group (CG) was obtained from nonexercised mice and is represented by the horizontal dashed line. One-way ANOVA indicated within-group and between-group differences. (*) Significantly different from CG; (a) Significantly different from other moments in the same group; (b) Significantly different from 0 h in the same group; (c) Significantly different from 6 h within the same group; (Φ) Significantly different from HIC at the same time; (#) Significantly different from the other groups at the same time; (*p* < 0.05).

The *Nos2* gene expression exhibited a trend to increase in the LIC group postexercise, but without statistical significance when compared to the CG or in any within-group comparisons. The HIC group exhibited a peak of *Nos2* gene expression immediately after the exercise bout, which was significantly higher than the CG, as well as all other groups at the same time. It was also higher than all other measures from the same group. On the other hand, in the HII group, a peak of *Nos2* gene expression was observed at 6 h postexercise, with significantly higher levels than the CG. However, at 12 h postexercise, the gene expression was repressed, exhibiting significantly lower levels than those observed at 6 h from the same group. Figure 8A presents the results from the *Nos2* gene expression.

*Nfe2l2* gene expression in the LIC group exhibited a trend to increase with time, achieving significantly higher levels than the CG at 24 h after the exercise bout, as well as higher than the HIC group at the same time. The gene expression level at 24 h was also higher than all other measures from the same group. *Nfe2l2* gene expression in the HIC group exhibited a divergent pattern from the other groups, with a peak of gene expression immediately after the exercise bout, which was significantly higher than the CG as well as higher than all other exercised groups at the same time. Additionally, the gene expression level at 0 h was also higher than all other measures from the same group. In the HII group, the pattern of *Nfe2l2* gene expression was similar to that observed in the LIC group, except for the presence of two peaks of gene expression at 6 h and 24 h postexercise. However, only the peak observed at 24 h was significantly higher than the CG, as well as higher than the HIC group at the same time, and higher than all other measures from the same group. Figure 8B presents the results from the *Nfe2l2* gene expression.

*Gpx1* gene expression exhibited a divergent trend among the studied groups. In the LIC group, the *Gpx1* gene expression exhibited a trend to increase at 6 h and repression at 12 h after the exercise bout, but without statistical significance (*p* > 0.05). The HIC group exhibited a peak of gene expression immediately postexercise, which was significantly higher than the CG, as well as higher than all the other exercised groups at the same time. Additionally, the gene expression level at 0 h was also higher than all other measures from the same group (*p* < 0.05). On the other hand, the HII group exhibited a peak of gene expression at 24 h postexercise, which was significantly higher than the CG, as well as higher than all other exercised groups at the same moment (*p* < 0.05). Figure 8C presents the results from the *Gpx1* gene expression.

## 4. Discussion

This study investigated the acute effect of different physical exercise protocols on redox balance and the gene expression of redox-related and mitochondrial-dynamics-related proteins in mice. Our results demonstrated that: (1) Blood lactate immediately postexercise was higher in HII exercise than in other studied exercises, while it was higher in HIC exercise than in LIC exercise; (2) Lipid peroxidation increased immediately postexercise in all groups, declining along the following 24 h, but remaining higher in the HII group and the HIC group than in the LIC group up to 6 h after exercise. Only the HIC group exhibited higher TBARS levels up to 24 h postexercise; (3) Antioxidant activity was higher at 24 h postexercise in all groups, but GPx activity was higher in the HII group than in the LIC and HIC groups at 0 h and 24 h after the exercise bout, respectively; (4) CS activity increased immediately postexercise in all groups, declining along the following 24 h. The HIC group exhibited a higher CS activity than the LIC group 12 h after the exercise bout, while the HII group exhibited higher CS activity than the LIC group immediately postexercise, and higher activity than all other groups at measures from 6 h to 24 h after exercise; (5) Mitochondrial-dynamics-related gene expression indicated a trend to increase mitogenic events in all exercised groups, with a very similar gene expression behavior for *Ppargc1α* in the HII and LIC groups, despite the opposite exercise characteristics. The mitochondrial-fusion-related gene expression trends toward an increase from 0 h to 24 h after the LIC group’s exercise bout, while the HIC and HII groups exhibited an inverse trend, with peaks at 0 h and then declining 24 h after the exercise bout. Gene expression related to fission and mitophagy exhibited a clear trend to repression in the HII group, but with some peaks of gene expression in the HIC group; and (6) Redox-related gene expression indicated a peak of *Nos2* and *GPx1* gene expression only in the HIC and HII groups, but at different times after the exercise bout, while *Nfe2l2* gene expression exhibited a peak in all groups, but immediately postexercise in the HIC group and 24 h after in the LIC and HII groups.

Lactate is recognized as an important metabolic intermediate, impacting the energy substrate utilization, cell signaling, and adaptation [17,18], especially mitochondrial and antioxidant adaptation [17,19,20]. Indeed, the mitochondrial lactate oxidation complex (mLOC) seems to be involved in the lactate-induced mitochondrial and redox adaptations, since it potentiates the mitochondrial lactate metabolism, increasing ROS production [19,21,22]. In this context, it is worthwhile to highlight that the mitochondrial lactate metabolism can generate ROS, specifically H_2_O_2_ [21,23], which could help to explain the greater TBARS concentration, a biomarker of lipid peroxidation, observed in our high-intensity groups (i.e., HIC and HII) that exhibited significantly higher blood lactate when compared to the LIC group.

H_2_O_2_ is cytotoxic at high concentrations, but its role as an important regulator of eukaryotic signal transduction has been established in recent years [24]. Our results indicated a TBARS concentration maintained for 24 h in animals from the HIC group, suggesting a harmful state sustained for a long time after a single exercise bout. Interestingly, animals from the HII group which were exposed to a higher load, but intercepted by short rest intervals, exhibited greater TBARS concentrations up to 12 h postexercise, and returned to the PRE exercise status between 12 and 24 h postexercise. These facts suggest that the use of simple rest intervals could minimize oxidative-induced tissue damage after a high-intensity-exercise bout. As high-intensity exercise leads to greater metabolic demands in a relatively short time, and the mitochondria represent an important ROS source, greater mitochondrial damage could be expected, owing to the oxidative stress overcoming the antioxidant defense capacity.

Indeed, considering the temporal profile of the CS activity from the HII group, the animals from the HIC group presented greater mitochondrial damage, since the HII group exhibited significantly greater CS activity 24 h after the exercise bout. Since the CS activity is commonly used as a marker of mitochondrial abundance within the tissue/cell [15], it is plausible to suggest that a positive mitochondrial adaptation was induced along with the 24 h interval after the HII-exercise bout, but blunted after the HIC-exercise bout, owing to the excessive oxidative damage. We hypothesize that the accumulation of mitochondrial damage in the HIC group could lead to dysfunctional mitochondria, which is characterized by the failure to convert the nutrient flux (e.g., fatty acid) efficiently into energy (ATP), but to overproduce ROS due to electron leakage to molecular oxygen [25].

The higher CS activity observed immediately and 6 h after all exercise bouts (i.e., LIC, HIC, and HII) is expected, owing to the greater exercise-induced metabolic demands. The significantly higher CS activity from the HII group sheds light on the oxidative demands of high-intensity exercises carried out for short intervals [26,27], since the capacity to maintain the ATP resynthesis reduces ~35% after the 10th second of maximal effort; on the other hand, a ~27% increase in the contribution of aerobic metabolism is estimated at the same time interval [27], and we used fourteen 20 s high-intensity efforts in the HII-exercise bout, which may justify the greater CS activity along the first hours postexercise.

Our results indicated that *Ppargc1α* gene expression increases along with the first hours after the exercise bout, with a peak observed 6 h after the exercise in all three groups. Curiously, the LIC and HII groups, with two protocols completely divergent in terms of intensity, duration, and execution mode, exhibited a similar behavior of *Ppargc1α* gene expression, with a peak at 6 h, followed by a decrease at 12 h, when compared to the peak, and a subsequent trend to increase again at 24 h after the exercise bout. The design of the LIC-exercise bout is, theoretically, more adequate than the other studied groups to induce endurance adaptations, since it involves low-intensity efforts sustained for a longer period. However, our results from the HII group corroborate previous studies that demonstrated short-term (e.g., two weeks or six sessions) endurance adaptations with HII exercise training [28]. Then, the use of intermittent design in high-intensity exercise seems to modulate the metabolic demands and ROS generation so that the immediate molecular response pattern from *Ppargc1α* gene expression was very close to the LIC-exercise bout. 

Previous studies have shown that lactate positively regulates the *Ppargc1a* gene expression [19,20], which could explain the peak of *Ppargc1α* gene expression immediately after the HIC exercise, but which was not observed after the HII exercise, which exhibited the highest blood (lactate) levels postexercise. Notwithstanding, we speculate that the HIC exercise induced greater mitochondrial damage immediately, which unfortunately was not measured in our study, leading to an immediate response aiming to restore the mitohormesis [29,30]. The exposed hypothesis could be corroborated by the immediate increase in the expression of genes involved in mitophagy (*Park2*), as will be discussed here.

The peak of *Ppargc1α* gene expression observed immediately and 6 h after the HIC-exercise bout could be related to the higher oxidative stress associated with this protocol, which applied a high-intensity effort sustained for 15 min. The increase in sarcoplasmatic (Ca^++^) induced by muscle contraction leads to the calcium/calmodulin complex and CREB activation, promoting the transcription of the PGC-1α protein gene [31]. Considering the impairment of Ca^++^ homeostasis at the sarcoplasmic reticulum level under sustained high-intensity efforts, characterized by increased release and the reduced reuptake of this ion [31], it can be suggested that the HIC protocol induced greater Ca^++^ ion homeostasis impairment, potentiating the stimulus for the *Ppargc1α* gene expression immediately after the exercise.

It is now known that mitochondrial morphologic changes are closely related to their function, stability, and turnover, and two events are relevant in this context: mitochondrial fusion and fission [4]. Mitofusin 2 is a key protein involved in mitochondrial fusion, leading to a hyperfused network to counteract metabolic insults, preserve cellular integrity, and protect against autophagy [32]; then, the observed increase of *Mfn2* gene expression in all studied groups indicated an immediate response to achieving metabolic demands from each exercise protocol. Curiously, the HIC protocol induced only one peak in the *Mfn2* gene expression, observed immediately postexercise, while the LIC and HII protocols exhibited an increase in the *Mfn2* gene expression but with opposite behaviors, with an immediate and significantly sustained increase up to 6 h after the exercise bout in the HII protocol, while the LIC protocol exhibited a progressive increase of the *Mfn2* gene expression, achieving significant values only 24 h after the exercise bout.

Mitochondrial fission is a physiological process necessary for two aims: (1) efficient mitophagy to eliminate damaged mitochondria, and/or (2) to ensure adequate mitochondrial mobility and distribution along with the cell [4,29]. Our results demonstrated a significant increase in the *Dnm1l* gene expression 6 h after the LIC-exercise bouts and 6 h and 12 h after the HIC-exercise bouts, while there was significant repression of this gene expression immediately and 12 h after the HII-exercise bout. These data could indicate a molecular sign to maintaining the hyperfused network, aiming to achieve the energetic demands imposed by the HII exercise. Since the the LIC-exercise bout is characterized by lower oxidative stress and metabolic demands along the workout, when compared to the high-intensity protocols, it is suggested that the increased *Dnm1l* gene expression observed 6 h after the LIC protocol is probably due to a physiological turnover process involved in mitochondrial dynamics, favoring the adequate mitochondrial mobility and distribution along the cell. On the other hand, the increase of the *Dnm1l* gene expression observed 6 h and 12 h after the HIC-exercise bout could be associated with greater mitochondrial damage, signaling for mitophagy needs, which seem to be corroborated by the *Park2* gene expression, as discussed in the following.

Parkin accumulation on the mitochondrial membrane has been reported during high-intensity exercises [4,33]. In our study, the HIC-exercise protocol was unique to induce a significant increase in the *Park2* gene expression, which could be associated with greater mitochondrial damage, leading to a molecular response directed to mitophagy, especially when the results from the *Dnm1l* gene expression are considered. Notwithstanding, the HII-exercise bout induced an opposite *Park2* gene expression than the HIC-exercise bout, suggesting that the inclusion of short intervals could avoid greater mitochondrial damage. In addition, the repression of *Park2* gene expression observed after the HII protocol could indicate molecular signaling to maintain/improve the hyperfused network, aiming to achieve the energetic demands of the high-intensity workout.

It is important to highlight that our data refer to an acute response to one exercise bout (i.e., after 24 h) and the effect of chronic exercise training on mitophagy may differ from an acute exercise bout, since some studies have demonstrated a decrease in mitophagy flux after an endurance-training period [4,34,35]. Indeed, the effect of training on mitochondrial dynamics is not always predictable, since some studies have demonstrated that chronic exercise promotes stimuli for fusion and fission in the same direction, suggesting that fission may be required to reorganize the mitochondrial network in response to metabolic demand [4].

Mitochondrial ROS is directly implicated in mitochondria-to-nucleus signaling, regulating the expression of enzymes involved in oxidative detoxification, which is part of the concept called mitohormesis [36,37]. This form of mitochondrial retrograde signaling involves a mitochondrially derived signal inducing alterations in nuclear gene expression [36], including the *Nfe2l2* gene [38].

As expected, all studied exercise protocols induced an increase in the *Nfe2l2* gene expression, with an immediate increase after the HIC protocol, but a progressive increase was observed after the LIC and HII protocols. Curiously, despite the very divergent in terms of intensity, duration, and execution mode, the LIC and HII protocols induced a peak of *Nfe2l2* gene expression 24 h after the exercise bout, indicating a similar molecular response involving this main regulator of cytoprotection, since Nrf2 regulates the expression of more than 200 cytoprotective genes [39].

GPx is an important peroxidase and exhibited a relatively low Km value for H_2_O_2_, suggesting effective removal of H_2_O_2_ at low substrate concentrations [40]. This fact suggests that GPx could contribute to situations of greater oxidative stress induced by H_2_O_2_ increases, as during exercise bouts, but is more important for homeostasis maintenance in daily situations. Curiously, *GPx1* gene expression was significantly greater only after the high-intensity protocol (i.e., HIC and HII), which could have occurred as a molecular response to deal better with the long-term oxidative stress induced by high-intensity exercises, since TBARS was significantly higher in the HIC and HII groups than in the LIC group in the first hours after the exercise bout. In addition, GPx activity was greater in the muscles from animals submitted to the HII protocol 24 h after the exercise bout, coinciding with the greater GPx1 gene expression in the same sample. It could indicate the increase of gene expression from 12 to 24 h after an exercise bout, with an effective translation and protein expression in the same period.

It is well established that oxidative stress is an important event to promote exercise-induced adaptations, and H_2_O_2_ seems to place a central role in this process. Despite this, nitric oxide also exhibits an important role, since it is involved in oxidative-induced adaptations, as well as promoting vascular modulation, inducing vasodilatation and angiogenesis [41]. The *NOS2* gene expression after the studied exercise protocols indicate a peak immediately after the HIC protocol and 6 h after the HII protocol, followed by repression 12 h after the HII protocol. The LIC protocol exhibited a trend to increase the *NOS2* gene expression in the first 24 h postexercise, but without statistical significance. These results indicate a divergent *NOS2* gene expression response among the studied exercise protocols, but with an evident trend to increase 24 h after all exercise protocols, which could contribute to exercise-induced microvascular adaptations, despite the sporadic repression of gene expression 12 h after the HII protocol, which was previously preceded by a peak and returned to the increasing trend 24 h after the exercise bout. Further studies should investigate deeply the mechanisms involved and the consequences of this exercise-induced *NOS2* gene expression increase, since the excessive nitric oxide production can stimulate apoptosis [42].

Although a pro-oxidant environment is important for the proper endoplasmic reticulum (ER) function, maintaining a high pro-oxidant status at the cellular level can compromise the adequate protein synthesis, which seems to involve the lack of calcium ion homeostasis both at the ER and sarcoplasmatic reticulum [43]. Additionally, a large load of proteins in the ER increases the probability of the folding process, accumulating unfolded proteins [44]. This event, called the unfolded protein response (UPR), leads to the attenuation of the translation rate, aiming to reduce the entry of proteins into the ER. Thus, the maintenance of the highly pro-oxidant state observed in the animals submitted to the HIC protocol may have impaired the acute response to the exercise, limiting the translation process, and, therefore, the effective synthesis of proteins involved in cytoprotection and mitochondrial dynamics.

## Figures and Tables

**Table 1:** Description of target genes involved in mitochondrial dynamics using the TaqMan^®^ Fast Advanced Master Mix system.

Gene	Identification	Reference Code	Protein
*Gapdh*	Mm99999915_g1	4453320	GAPDH
*Ppargc1α*	Mm01208835_m1	4453320	PGC1-α
*Mfn2*	Mm00500120_m1	4453320	MFN2
*Dnm1L*	Mm01342903_m1	4448892	Drp1
*Park2*	Mm01323528_m1	4448892	Parkin
*Nfe2l2*	Mm00477784_m1	4453320	NRF-2
*Gpx1*	Mm00656767_g1	4453320	GPx1
*Nos2*	Mm00440502_m1	4453320	NOS-2

## Data Availability

The data used to support the findings of this study are included within the article.
